# Changes in the Species and Functional Composition of Activated Sludge Communities Revealed Mechanisms of Partial Nitrification Established by Ultrasonication

**DOI:** 10.3389/fmicb.2022.960608

**Published:** 2022-07-19

**Authors:** Yu Xue, Min Zheng, Shuang Wu, Yanchen Liu, Xia Huang

**Affiliations:** ^1^State Key Joint Laboratory of Environment Simulation and Pollution Control, School of Environment, Tsinghua University, Beijing, China; ^2^Australian Centre for Water and Environmental Biotechnology, The University of Queensland, St. Lucia, QLD, Australia

**Keywords:** wastewater treatment, partial nitrification, microbial community, metagenomics, ultrasonic treatment

## Abstract

To achieve energy-efficient shortcut nitrogen removal of wastewater in the future, selective elimination of nitrite-oxidizing bacteria (NOB) while enriching ammonia-oxidizing microorganisms is a crucial step. However, the underlying mechanisms of partial nitrification are still not well understood, especially the newly discovered ultrasound-based partial nitrification. To elucidate this issue, in this study two bioreactors were set up, with one established partial nitrification by ultrasonication while the other didn't. During the operation of both reactors, the taxonomic and functional composition of the microbial community were investigated through metagenomics analysis. The result showed that during ultrasonic partial nitrification, ammonia-oxidizing archaea (AOA), *Nitrososphaerales*, was enriched more than ammonia-oxidizing bacteria (AOB), *Nitrosomonas*. The enrichment of microorganisms in the community increased the abundance of genes involved in microbial energy generation from lipid and carbohydrates. On the other hand, the abundance of NOB, *Nitrospira* and *Nitrolancea*, and Comammox *Nitrospira* decreased. Selective inhibition of NOB was highly correlated with genes involved in signal transduction enzymes, such as encoding histidine kinase and serine/threonine kinase. These findings provided deep insight into partial nitrification and contributed to the development of shortcut nitrification in wastewater treatment plants.

## Graphical Abstract

Schematic diagram of partial nitrification mechanism. Ultrasonic treatment was applied here as a selection force to change the microbial community. In contrast to the previous perception that AOB and NOB are the main players of partial nitrification, we found AOA played a dominant role. Genes encoding enzymes involved in signal transduction, energy conversion and lipid metabolism were also significantly altered and associated with changes in the microbial community during this process. 
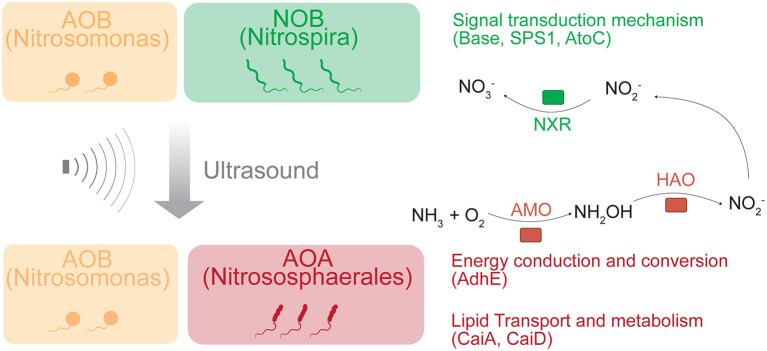

## Introduction

Aerobic nitrification is an important biological process for nitrogen removal from wastewater. It conventionally takes place in two steps. First, ammonia is oxidized to nitrite mainly by ammonium-oxidizing bacteria (AOB). Second, nitrite is oxidized to nitrate by nitrite-oxidizing bacteria (NOB) (Kuenen, 2008). Both steps require large amounts of dissolved oxygen, supplied by blowers that consumed a lot of energy in conventional wastewater treatment plants (WWTPs) (Gude, 2015). Following the second step of nitrification, nitrate needs to be further reduced to nitrogen gas by organic carbon as electron acceptors during anoxic denitrification. In this step, it's required to add external carbon such as ethanol and formic acid because organic carbon in municipal wastewater is always insufficient (Isaacs and Henze, 1995; Torresi et al., 2017). As mentioned above, the high demand for oxygen and carbon are the two main factors for the high energy consumption during the operation of WWTPs. In contrast, the shortcut nitrogen removal processes cease the conventional nitrification to the first step of producing nitrite, which is then used as an electron acceptor for denitrification or anammox (McCarty, 2018). This saves energy. More specifically, the shortcut processes reduce aeration consumption by 25% in the nitrification step and save 40 and 100% of the organic demand for the subsequent denitrification and anammox processes, respectively (Verstraete and Philips, 1998; Gilbert et al., 2014).

Implementation of shortcut nitrogen removal requires constant inhibition of nitrite oxidation while maintaining ammonia oxidation, which has been considered difficult to achieve in the past decades. To achieve partial nitrification, various approaches have been proposed, including the control of concentrations of substrates, such as dissolved oxygen (Blackburne et al., 2008), and the introduction of inhibitory substances such as free ammonia (FA) (Wang et al., 2017), free nitrous acid (FNA) (Wang et al., 2014) and sulfide (Seuntjens et al., 2018). In recent years, it has been discovered that the introduction of external energy can also achieve partial nitrification. For example, ultrasound, an acoustic energy wave with a frequency above 20 kHz which is often used to reduce excess sludge in wastewater treatment plants (Wu et al., 2018), has recently been found to enable shortcut nitrogen removal processes (Zheng et al., 2016; Huang et al., 2019, 2020).

For all these partial nitrification methods, the mechanism of ultrasound treatment seems to be more implicit than that of direct control of the substrate concentration or addition of inhibitory substances. Mathematical kinetic analysis of the activated sludge inferred that AOB activity increased due to the promotion of enzymatic catalytic reaction of the cells (Zheng et al., 2019). Carbonyl, hydroxyl and amine functional groups were observed in the analysis of extracellular polymeric substances (EPS) of the sludge during the sonication process, indicating a positive effect of ultrasonic treatment on the mass transfer efficiency of microorganisms (Tian et al., 2021). However, little is known about the actual micro mechanism of ultrasonic partial nitrification. To decipher this wastewater treatment process, the metagenomic method may provide some insights into the level of microbial community and functional composition. Take FNA-based partial nitrification as an example, by applying metagenomics and metaproteomics approach, the resistance of AOB was found to be associated with enzymes involved in oxidative stress and energy generation (Laloo et al., 2018).

Furthermore, an increasing number of nitrifying microorganisms have been found to be involved in nitrification in wastewater treatment plants. Some of them, such as comammox *Nitrospira* (Roots et al., 2019; Cotto et al., 2020), *Nitrotoga* (Zheng et al., 2020), and *Nitrolancea* (Wang et al., 2020), may contribute to the deterioration of partial nitrification. On the contrary, other nitrifying microorganisms such as ammonia-oxidizing archaea (AOA), may promote partial nitrification. Though the communities of NOB and AOB have been intensively studied in the past few years, the role of AOA in partial nitrification has not received much attention. In fact, the presence of AOA in wastewater has been confirmed in worldwide WWTPs (Limpiyakorn et al., 2013). AOA was also frequently found to be more abundant than AOB in many low ammonium concentration environments from soils to oceans (Offre et al., 2013), due to the higher substrate affinity of AOA than that of AOB (Martens-Habbena et al., 2009). Therefore, AOA instead of AOB may play an important role in partial nitrification, especially in the latest development of mainstream wastewater treatment, which has a much lower ammonium concentration (30–100 mgN/L) (Metcalf et al., 1991) than the well-studied side-stream wastewater (500–1,000 mgN/L) (Lackner et al., 2014). In addition to AOB and AOA, the inhibition mechanisms of the NOB community are not fully understood either, which makes it difficult to avoid the deterioration of partial nitrification due to the variation of the NOB community. To deepen our understanding of partial nitrification, metagenomic analysis allows us to obtain a comprehensive description of the taxonomic and functional composition of the community.

This study aimed to reveal the taxonomic functional characteristics of the microbial community during partial nitrification. Initially, two bioreactors, reactor C and reactor T reactors were set up and operated under the same condition to reach the same full nitrification. Afterward, reactor T was treated by ultrasound to transit from full nitrification to stable partial nitrification. During 268 days of operation of the two reactors, time series samples were regularly collected to analyze microbial community and functional changes using metagenomic analysis, 16S rRNA gene amplicon sequencing, and archaea *amoA* gene amplicon sequencing. Through analysis of the community, the relationship between microorganisms and environmental parameters was deduced, in order to understand the mechanism of selective inhibition of nitrifying microorganisms during the establishment of partial nitrification.

## Materials and Methods

### Activated Sludge Sampling

To compare partial nitrification and full nitrification, two sequencing batch reactors (reactor C and reactor T) with a volume of 9 L each were set up in this study. The influent of the reactors was real municipal wastewater with the component listed in Supplementary Table S1. Both reactors were operated for two cycles per day. Each 12-h cycle consisted of a 13-min feeding period when 4 L of wastewater was pumped into the reactor, a 2.5-h anoxic mixing period, a 5.5-h aerobic aeration period, a 50-min settling period, a 10-min decanting period, and a 167-min resetting period.

The inoculated sludge came from a pilot-scale aerobic membrane bioreactor that treats domestic wastewater and provides 1,200–1,300 tons/day of reuse water using the A/O process. The removal rates of organic matter and ammonia nitrogen of the seed sludge were above 90%. Both reactors in our study were operated at room temperature of 25°C. The SRT was kept at 30–40 days by a regular discharge of activated sludge. The dissolved oxygen (DO) was monitored in real time and maintained above 0.8 mg/L during the aerobic period. The pH of influent and effluent was 7.0–8.0.

**Figure 1:**
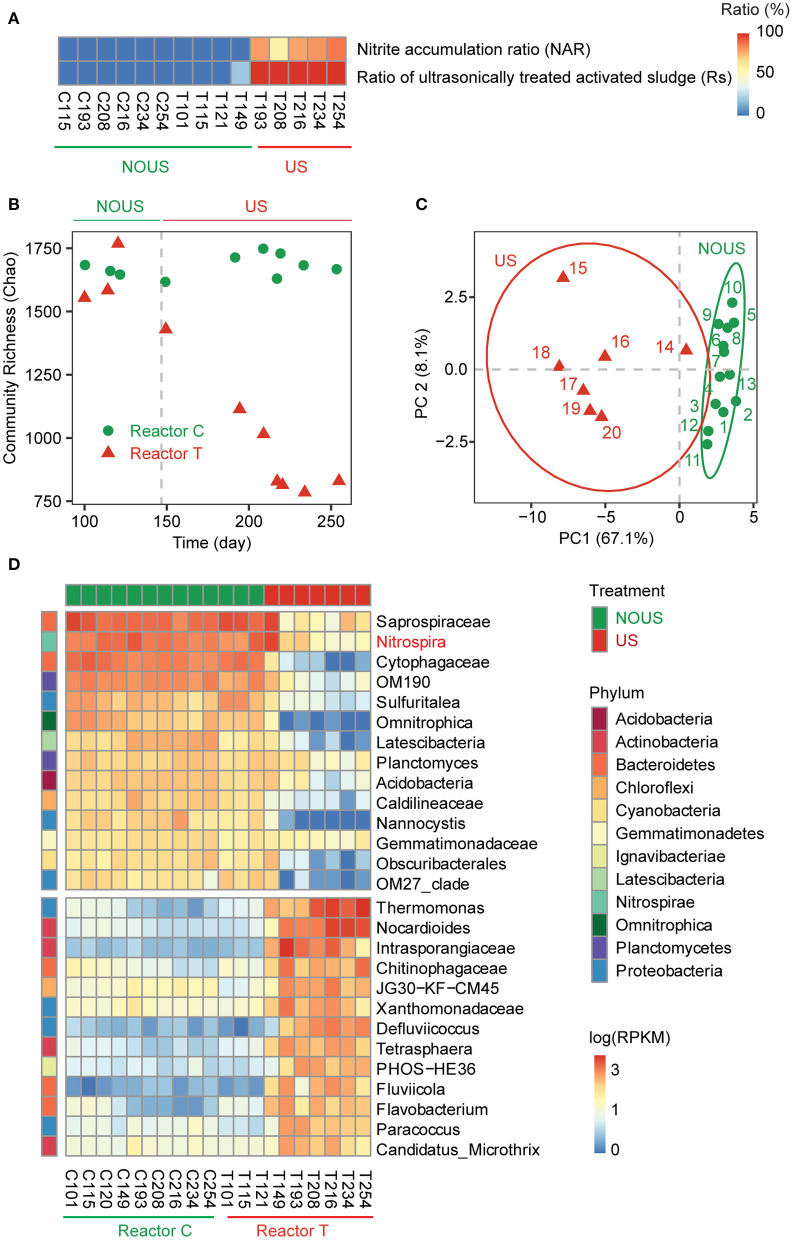
Phylogenetic profile of the microbial community. **(A)** Performance of reactor. **(B)** Variation of community diversity with reactor operation time using Chao index. **(C)** Principle component analysis (PCA) of the microbial community. **(D)** Heatmap of the genera contributing to the principal components of the microbial community and their abundance in each sample. The abundance of microbes was scaled to log10 of reads per kilobase per million mapped reads (RPKM). Samples were clustered into ultrasonic treatment (US) and without ultrasonic treatment (NOUS) groups by using PCA and ANOSIM. The phylum level of each genus was labeled on the left of the heatmap.

Once stable and full nitrification was achieved in both reactors, a certain ratio of sludge (Rs in Figure 1A) was collected from reactor T in the resetting period and then treated with ultrasonic waves using an ultrasonic generator (ZJS−1,000–500, Hangzhou Success Ultrasonic Co., Ltd.) with a fixed frequency of 40 kHz and power of 100 W. The energy density of ultrasonic treatment (Es, kJ/ml) was calculated as follows.


$$\begin{array}{l} Es=\;\frac{P\times t}{V} \end{array}$$

*P* is the power of ultrasound (W), which was 100 W in this study. *t* is the duration of ultrasound treatment (s), which was listed in Table 1. *V* is the volume (L) of activated sludge collected from the reactor and thickened to 10–12 g MLSS/L for ultrasonication, which was 0.1 L in this study. The calculated Es was listed in Table 1.

**Table 1:** Reactor performance, ultrasonic treatment conditions, and sampling.

**No**.	**Time (d)**	**Sample name**	**Specific** **ammonia** **oxidation rate** **(mg N/(h·g** **MLSS)**	**Influent** **ammonium** **concentration** **(mg N/L)**	**Energy density** **of ultrasound** **(kJ/mL)**	**Ultrasonication** **time (min)**	**Group**	**Metagenome** **sampling**
**Reactor C**								
1	101	C101	4.25	53.92	0	0	NOUS^a^	-
2	115	C115	4.25	33	0	0	NOUS	√
3	121	C121	4.36	31.43	0	0	NOUS	-
4	149	C149	4.36	37.19	0	0	NOUS	-
5	193	C193	4.4	54.1	0	0	NOUS	√
6	208	C208	4.4	48.18	0	0	NOUS	√
7	216	C216	4.4	81.74	0	0	NOUS	√
8	220	C220	4.4	81.74	0	0	NOUS	-
9	234	C234	4.4	61.51	0	0	NOUS	√
10	254	C254	4.4	25.71	0	0	NOUS	√
**Reactor T**								
11	101	T101	6.09	53.92	0	0	NOUS	√
12	115	T115	6.09	33	0	0	NOUS	√
13	121	T121	4.78	31.43	0	0	NOUS	√
14	149	T149	4.78	37.19	0.6	10	NOUS	√
15	193	T193	5.95	54.1	0.9	15	US^b^	√
16	208	T208	5.95	48.18	0.9	15	US	√
17	216	T216	5.95	81.74	0.9	15	US	√
18	220	T220	5.95	81.74	0.9	15	US	-
19	234	T234	5.95	61.51	0.9	15	US	√
20	254	T254	5.95	25.71	0.9	15	US	√

a *Group of low ultrasonic treatment and no ultrasonic treatment (NOUS) after clustering samples using PCA and ANOSIM.*

b *Group of ultrasonic treatment (US) after clustering samples using PCA and ANOSIM*.

Ammonia oxidation rate [mg N/(h·g MLSS)], an index representing the activity of AOB, was calculated during the aerobic period as the slope of ammonium concentration (mg N/L) vs. time (h) and then divided by the biomass concentration (g MLSS/L). Nitrite accumulation ratio (NAR, %), an index used to evaluate the efficiency of partial nitrification (Gu et al., 2012), was calculated as follows.


$$\begin{array}{l} NAR=\;\frac{\{NO_{2}^{-}\}\;}{\{NO_{2}^{-}\}\;+\{NO_{3}^{-}\}\;} \end{array}$$

Where {${\mathrm{NO}}_{2}^{-}$} and {${\mathrm{No}}_{3}^{-}$} is the effluent concentration of ${\text{NO}}_{2}^{-}$ (mg N/L) and ${\text{NO}}_{3}^{-}$(mg N/L), respectively.

During the operation of both reactors, a total of 20 activated sludge samples were collected for further microbiological analysis. First, the sludge samples were centrifuged at 5,000 rpm for 4 min, then the supernatant was removed and replaced with an equal amount of ethanol. The collected samples were all stored in a refrigerator at −80°C for DNA extraction.

### DNA Extraction and 16S rRNA Gene Amplicon Sequencing

DNA was extracted from the samples using the FastDNA SPIN Kit for Soil (MP Biomedicals, LLC, Solon, OH, USA) kit according to the manufacturer's protocols. After extraction, the DNA was finally resuspended with ultrapure water. The concentration and purity of the DNA were determined by NanoDrop 2000 UV-vis spectrophotometer (Thermo Scientific, Wilmington, USA). The quality of DNA was also checked by 1% agarose gel electrophoresis.

All 20 DNA samples were used for 16S rRNA gene sequencing. The V4-V5 hypervariable regions of the bacteria 16S rRNA gene were amplified using primer pair of 515F (5′ -GTGCCAGCMGCCGCGG-3′) and 907R (5′ -CCGTCAATTCMTTTRAGTTT-3′) in a PCR system (GeneAmp 9700, ABI, USA). The PCR mixture was prepared in triplicate, each in a volume of 20 μL, containing 4 μL of 5 × FastPfu Buffer, 2 μL of 2.5 mM dNTPs, 0.8 μL of each primer (5 μ M), 0.4 μL of FastPfu Polymerase and 10 ng of template DNA. The PCR reactions were performed according to the following procedure: denaturation at 95°C for 3 min, 27 cycles of which each contains 30 s at 95°C, 30 s at 55°C for annealing and 45 s at 72°C for elongation and a final extension at 72°C for 10 min. The PCR products were then purified using 2% agarose gel and AxyPrep DNA Gel Extraction Kit (Axygen Biosciences, Union City, CA, USA) and were quantified using QuantiFluor^™^-ST (Promega, USA). Finally, the purified amplicons were pooled in an equimolar and paired-end sequenced (2 ×300) on an Illumina MiSeq platform (Illumina, San Diego, USA).

The raw reads obtained from 16s rRNA gene sequencing were quality controlled using Fastp 0.19.6 (Chen et al., 2018) and Flash 1.2.11 (Magoč and Salzberg, 2011) with the following criteria: (1) Any site with an average quality score below 20 in a 50 bp sliding window was truncated; (2) Reads with more than two nucleotide mismatches with primers and those containing ambiguous bases were removed; (3) Overlaps longer than 10 bp were merged. Then, the reads with similarity above 97% were clustered into operational taxonomic units (OTUs) using Uparse 7.0.1090 (Edgar, 2013). The taxonomy of the 16S rRNA gene sequence was identified using the RDP classification algorithm (Wang et al., 2007) and Silva (SSU 138.1) 16S rRNA database (Quast et al., 2012) with a confidence threshold of 70%.

### Metagenomic Sequencing, Assembly, and Binning

Fifteen DNA samples were selected for metagenomic sequencing. DNA library was constructed using NEXTflex Rapid DNA-Seq Kit (Bioo Scientific, Austin, TX, USA) in Covaris M220 System to produce ~400 bp DNA, which was sequenced in the Illumina Hiseq 2000 system using NovaSeq Reagent Kits. The raw reads obtained from metagenomic sequencing were quality controlled using Seqprep (St. John, 2016) to cut off adaptor sequences at 3′ and 5′ end and Sickle 1.33 (Joshi and Fass, 2011) to remove reads that were <50 bp in length, had a quality score below 20, and contained N base. High-quality reads from each sample were then assembled into contigs using Multiple_Megahit (Dinghua et al., 2015) with default parameters of IDBA-UD (Peng et al., 2012), Megahit (Li et al., 2016) and Newbler (Margulies et al., 2005). MetaGene (Noguchi et al., 2006) was used to predict open reading frame (ORF) from contigs. Sequences with nucleic acid length >100 bp were translated into amino acid sequences, resulting in an ORF set containing 30,340,623 ORFs. The sequences with nucleotide identity >95% and coverage above 90% were clustered together using CD-HIT (Fu et al., 2012). The longest ORF of each cluster was then taken as a representative sequence to construct a non-redundant gene set containing 13,364,656 genes. For metagenomic binning, we first combined the reads of all samples and then assembled them into contigs using MEGAHIT (Li et al., 2016), which constructed the De-Bruijn graph based on the overlap between k-mers. The contigs with more than 800 bp were then used for binning by VAMB (Nissen et al., 2021) to get MAGs. The completeness, contamination, GC ratio and genome size of draft MAGs were estimated using CheckM (v.1.0.18) (Parks et al., 2015). The ORFs of the MAGs were predicted using Prodigal (v. 2.6.3) and default parameters (Hyatt et al., 2010). Then, the MAGs with completeness above 50% and contamination below 10% were used for further taxonomy classification and abundance estimation. The taxonomic and functional annotation of MAGs and genes was conducted using Diamond (Buchfink et al., 2015) and Blast (Altschul et al., 1997) with an E-value of 1E-5. The databases used for annotation included the Non-Redundant Protein Sequence Database (Pruitt et al., 2007) for taxonomy annotation, EggNOG database (Lars Juhl et al., 2008) for Clusters of Orthologous Groups (COGs) annotation, KOBAS 2.0 database (Chen et al., 2011) for Kyoto Encyclopedia of Genes and Genomes (KEGG) annotation, and NCyc database (Tu et al., 2018) for nitrogen cycle genes annotation.

To calculate the abundance of genes and MAGs in every sample, high-quality reads were aligned with genes and MAGs with 95% identity as the threshold using Bowtie 2 (Langmead and Salzberg, 2012). The abundance of genes and MAGs were estimated based on reads per kilobase per million mapped reads (RPKM) (Mortazavi et al., 2008; Lawson et al., 2017; Armstrong et al., 2018; Pushkarev et al., 2018; Kim et al., 2021) values of metagenomic reads that mapped to each gene and MAG, where the read counts were normalized by the sequencing depth and gene length. The calculation formula of RPKM was expressed as follows (Mortazavi et al., 2008).


$$\begin{array}{l} RPKM=\;\frac{n_{r}}{\frac{N}{10^{6}}\;\times \frac{L}{10^{3}}}=\;\frac{10^{9}\;\times n_{r}}{N\;\times L} \end{array}$$

Where *n*_*r*_ is the number of reads mapped to the target gene, *N* is the total sum number of reads that effectively mapped to all genes in the sample, and *L* is the gene length. The above calculation method of RPKM solves the biases that the deeper the sequencing depth and the longer the gene length, the more reads are obtained from sequencing. After the normalization of reads, the relative abundance was calculated using the ratio of each RPKM value and the sum of the RPKM values of all genes and all species in each sample (Zhao et al., 2018).

### Archaeal *amoA* Gene Amplicon Sequencing

Amplification of archaeal *amoA* genes was performed using the primers Arch-amoAF (5′-STAATGGTCTGGCTTAGACG-3′) and Arch-amoAR (3′- GCGGCCATCCATCTGTATGT-5′) (Francis et al., 2005) on a thermal cycler (GeneAmp 9700 Applied Biosystems, USA). The samples were pre-amplified to ensure that the amplification product could reach a concentration that is appropriate for further sequencing. Out of the 15 samples, seven samples were not successfully pre-amplified, most likely due to insufficient abundance of AOA in the samples. For the eight samples successfully pre-amplified, PCRs of total archaeal *amoA* genes were performed using TransGen AP221-02:TransStart Fastpfu DNA Polymerase. The PCR program consisted of an initial denaturation step at 95°C for 3 min, followed by 37 cycles at 95°C for 30 s, 53°C for 30 s, and 72°C for 45 s, maintained at 72°C for 10 min, and a final annealing step at 10°C. PCR products were then purified using 2% agarose gel and AxyPrep DNA Gel Extraction Kit (Axygen Biosciences, Union City, CA, USA) and were quantified using QuantiFluor™-ST (Promega, USA). Finally, purified amplicons were pooled in equimolar and paired-end sequenced on the Illumina MiSeq platform (Illumina, San Diego, USA). The *amo*A gene of archaea recovered from amplification was aligned with 1,190 published archaeal *amoA* gene sequences with taxonomy information (Alves et al., 2018). The phylogenetic tree of these *amoA* gene sequences was built using Clustal Omega (Sievers et al., 2011).

### Statistical Analysis

To estimate the diversity of the microbial community, the Chao index (Chao, 1984) was calculated using Mothur 1.43.0 (Schloss et al., 2009). To cluster the abundance matrices of all samples, the unweighted pair group method with arithmetic mean clustering algorithm (UPGMA) was applied in Qiime (Caporaso et al., 2010). The distance of every two samples was calculated using the Bray-Curtis algorithm to generate a β diversity matrix for clustering. To identify the drivers for clustering, principal component analysis was performed in R with FactoMineR package (Lê et al., 2008), which could find the species and functions with high contribution to each principal component. To quantify and compare the contribution of each driver, we conducted LEfSe analysis (Segata et al., 2011), which firstly used the Kruskal-Wallis (KW) sum-rank test to identify species with significant abundance differences between groups, and then used linear discriminant analysis (LDA) to estimate the effect of the species or functions on the group difference. The group comparison strategy of LEfSe analysis was all-against-all (stricter than one-against-one), and *p* < 0.05 between groups were declared to be significantly different. To predict the relationship between the main phylogenetic and functional contributors, Spearman correlations were computed. The absolute value of Spearman correlations above 0.5 was transformed into links between two contributors in the network. The Networkx package (Hagberg et al., 2008) in Python was then used to construct network figures. Wilcoxon rank-sum tests were used to test specific species and functions that were significantly different between the groups. All the figures, including boxplot, scatter point plot, and bar chart, were plotted in R using the ggplot2 package (Zheng et al., 2016). Heatmap was plotted in R with pheatmap package (Kolde, 2015).

## Results and Discussion

### Performance of Reactor During Partial Nitrification

Initially, both reactors were operated under identical conditions and neither was ultrasonicated so that full nitrification was maintained till the two reactors reached similar specific ammonia oxidation rates on day 121 (Table 1). From day 122 to 183, to achieve partial nitrification, a low energy density of 0.6 kJ/ml ultrasound was tentatively introduced in reactor T. However, no stable partial nitrification was observed during this period, indicating that the ultrasound intensity was too low to effectively inhibit nitrite oxidation. On day 184, the energy density of ultrasound was raised up to 0.9 kJ/ml. Thereafter, partial nitrification was gradually established and the nitrite accumulation ratio (NAR) finally rose to 100% (Figure 1A), indicating that nitrite oxidation was inhibited more than ammonium oxidation. The inhibition of nitrite oxidation by ultrasound was within expectation, considering that ultrasound is commonly used to inactivate bacteria in pure culture experiments and to reduce excess activated sludge in wastewater treatment plants (Zhang et al., 2009; He et al., 2011). Nonetheless, ammonium oxidation was not inhibited by ultrasound in our study. In fact, the ammonia oxidation ratio in both reactors was above 95%, and the ammonia oxidation rate remained stable in ultrasonic samples, with an average value [5.95 mg N/(h·g MLSS)] even slightly higher than that of the samples without sonication [4.44 mg N/(h·g MLSS)]. A similar promotional effect of low-intensity ultrasound on ammonium oxidation was also reported in other metabolic pathways, such as hydrogen production (Yin et al., 2018), iron-sulfur cluster biosynthesis (Zhang et al., 2020), and ATP release (Belcik et al., 2017).

### Microbial Community Structure

To investigate what exactly happened to microorganisms in the reactor during the establishment of ultrasonic partial nitrification, the microbial community in both reactors was analyzed. In reactor C, the community diversity remained stable throughout the operation of the reactor, while in reactor T, the microbial community diversity decreased once ultrasound was introduced to build partial nitrification (Figure 1B). Other partial nitrification methods such as FA (Kinh et al., 2017), FNA (Wang et al., 2016) and low DO treatment (Zhang et al., 2019) also found a decrease in the community diversity.

Before delving into microbial community structure, we first examined the linear regression models (Supplementary Figure S1) of community composition derived from 16S rRNA gene sequencing and metagenomic sequencing (Supplementary Tables S2, S3). The results showed that the microbial community compositions developed from the two methods were similar (Supplementary Figure S1). In both reactors, Proteobacteria and Bacteroidetes were the two most abundant phyla (Supplementary Figure S2), which were also the two most abundant and the most frequently occurring phyla in global WWTPs (Wu et al., 2019), indicating that the microbial community structure of our samples was generally representative.

Among the eight most abundant phyla, all except Proteobacteria and Actinobacteria were decreased by ultrasound (Supplementary Figure S2). Principal component analysis (PCA) showed that all samples in our study formed two clusters (Figure 1C), exactly one cluster (US) was subjected to strong ultrasonication and the other cluster (NOUS) was not. There were 28 genera that contribute to this differentiation of microbial community structure (Figure 1D; Supplementary Figure S3). Among these genera, the enriched genera were from three phyla, while the reduced genera were from six phyla (Figure 1D). In other words, the reduced phyla were more diverse than the enriched phyla, suggesting that the microbial community appears to delete branches of the phylogenetic tree, making it more clustered during partial nitrification. Similar to the PCA result, the LDA result showed that the Proteobacteria phylum was significantly enriched by ultrasound, while the more phyla such as Chloroflexi, Planctomycetes, Nitrospirae and Acidobacteria decreased (Supplementary Figure S4). Other partial nitrification methods also showed similar microbial community clustering (Supplementary Table S4). AOB happens to belong to the Proteobacteria, the phylum that had the highest resistance to ultrasound (Supplementary Figures S2, S4). In contrast, NOB belongs to one of the many small branches of the phylogenetic tree that were removed during ultrasonic partial nitrification (Figure 1D).

### Ammonia-Oxidizing Bacteria and Ammonia-Oxidizing Archaea

**Figure 2:**
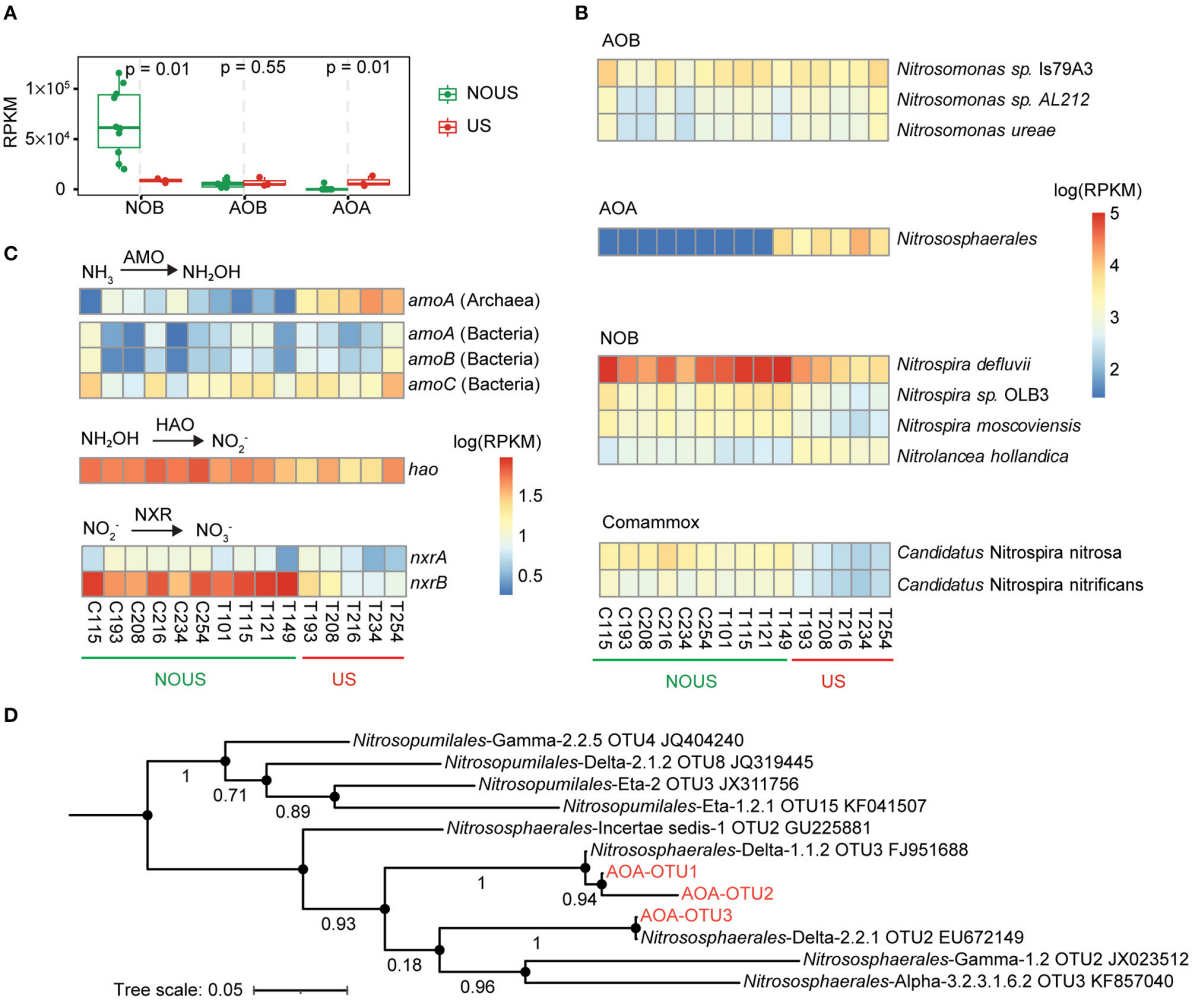
Microbes and genes involved in nitrification. **(A)** Comparison of NOB, AOB and AOA using Wilcoxon test. **(B)** Abundance variation of microorganisms involved in nitrification. The abundance of genes and microbes was scaled as reads per kilobase per million mapped reads (RPKM). **(C)** Changes in the abundance of genes encoding enzymes involved in nitrification, including ammonia monooxygenase (AMO), hydroxylamine oxidoreductase (HAO) and nitrite oxidoreductase (NXR). **(D)** Phylogenetic tree of archaeal *amoA* gene. Three archaeal *amoA* genes found in this study were aligned with 1,190 near-full-length (591–594 bp) archaeal *amoA* genes collected from pure culture and environmental enrichment to a build phylogenetic tree, of which nearest OTUs were selected to plot the above figure.

The relative abundance of AOB was 0.35% on average and was not decreased by ultrasonic treatment (Figure 2A, Wilcoxon test *p* > 0.05). The three AOB species that were not ultrasonically inhibited included *Nitrosomonas* sp. IS39A3, *Nitrosomonas* sp. AL212 and *Nitrosomonas ureae*, all of which belong to the genus *Nitrosomonas* (Figure 2B). In AOB, the oxidation of ammonia was accomplished in two steps. First, ammonia (NH_3_) is oxidized to hydroxylamine (NH_2_OH) *via* ammonia monooxygenase (AMO), and then NH_2_OH is simultaneously oxidized to nitrite (${\text{NO}}_{2}^{-}$) *via* hydroxylamine oxidoreductase (HAO) (Sayavedra-Soto and Arp, 2011). The *amoA, amoB* and *amoC* genes encode the α, β, and γ subunits of AMO, and the *hao* gene encodes HAO (Klotz and Stein, 2011). In our study, the abundance of bacterial *amoA, amoB*, and *amoC* genes remained stable along with the operation time of reactors (Figure 2B; Supplementary Figure S5, Wilcoxon test *p* > 0.05), confirming the high tolerance of AOB during ultrasonic partial nitrification.

The abundance of the archaeal *amoA* gene, a marker gene widely used to detect ammonia-oxidizing archaea (AOA) in the environment (Pester et al., 2012), increased significantly by ultrasonic treatment and exceeded the bacterial *amoA* gene by 5.7 times (Figure 2C; Supplementary Figure S5, Wilcoxon test *p* = 0.029). To reconfirm the presence of AOA, amplification of the *amoA* gene of Archaea was performed in all samples. Seven of the 15 samples were not successfully pre-amplified due to the low concentration of AOA without ultrasonic treatment. For the successfully pre-amplified samples, we sequenced and aligned the archaeal *amoA* gene sequences (Supplementary Table S5) with 1,190 nearly full-length sequences of the archaeal *amoA* gene (Alves et al., 2018). Phylogenetic analysis placed them close to the order *Nitrososphaerales* (Figure 2D). *Nitrososphaerales* are distributed in various soil and water environments worldwide and are the most frequently detected AOA (Alves et al., 2018).

AOA was reported to be not as widespread as AOB in WWTPs (Mußmann et al., 2011), which may explain why AOB was usually considered the main ammonium oxidizer in partial nitrification of wastewater. However, here in our study, the average abundance of the enriched AOA (0.73%) was higher than the average abundance of AOB (0.35%). The specific ammonium oxidation rate also increased with the enrichment of AOA (Table 1), suggesting that higher AOA abundance was translated into a higher ammonium oxidation rate of activated sludge in wastewater treatment. AOA is suitable for growth at low ammonia nitrogen concentrations (Martens-Habbena et al., 2009). In our study, the feedwater to both reactors was domestic wastewater with a low ammonium concentration of 46 mgN/L (Supplementary Table S1), rather than side-stream wastewater or industrial wastewater with a high ammonium concentration of 500 mgN/L (Lackner et al., 2014). Exposure to higher ammonia concentrations may expose AOA to earlier inhibition than AOB (Mußmann et al., 2011; Yapsakli et al., 2011; Sauder et al., 2012; Gao et al., 2016a). The future mainstream partial nitrification and anammox processes are oriented to wastewater with low ammonium concentration. Therefore, more attention needs to be paid to AOA in wastewater, especially in establishing and maintaining partial nitrification.

In addition to low ammonium concentration, ultrasonic treatment at the appropriately low energy intensity is also a factor to enrich AOA in our study (Figure 2). The major difference between archaea and bacteria is cell membrane composition. What's more, most archaea such as hyperthermophiles, halophiles and acidophiles, live in extreme ecological environments (Valentine, 2007). Faced with the extreme destructive energy of ultrasound, the advantage of archaea over bacteria in terms of biofilm could support the ultrasonic selection of AOA over AOB and NOB. For example, the ether bonds, which link the glycerol and hydrocarbon chain of the cell membrane of archaea, are more stable than the ester lipids of bacteria (Baba et al., 1999; Valentine, 2007). More specifically, this is because the R-COO-R of easter is less stable due to the nucleophilic attack of the carbonyl carbon, compared with the R-O-R of the ether (Baba et al., 1999).

### Nitrite-Oxidizing Bacteria

The total abundance of NOB species gradually decreased with the establishment of partial nitrification (Figures 2A,B). These NOB species mainly belonged to the phylum Nitrospirae, the most common NOB phylum in global WWTPs (Wu et al., 2019). Three species including *Nitrospira defluvii, Nitrospira* sp. OLB3, and *Nitrospira moscoviensis* were detected in this phylum (Figure 2B). Among all these NOB species, *Nitrospira defluvii* was the most abundant species in the whole community of this study, with an average relative abundance of 6.05% in the samples without ultrasonic treatment.

Another NOB species, *Nitrolancea hollandica* which belongs to the phylum Chloroflexi, was also detected. *Nitrolancea hollandica* was reported to tolerate high FNA and thus deteriorate partial nitrification (Wang et al., 2020; Yu et al., 2020). Similarly, in our partial nitrification reactor, the abundance of *Nitrolancea hollandica* increased a little under high energy density ultrasonication (Figure 2B; Supplementary Figure S6, Wilcoxon test *p* < 0.05). In spite of that, the abundance of *Nitrolancea hollandica* (1,325 RPKM) ended up being much lower than that of *Nitrospira defluvii* (6,038 RPKM), suggesting that *Nitrolancea hollandica* played a minor role in weakening ultrasonic partial nitrification.

Comammox *Nitrospira* species including *Candidatus* Nitrospira nitrosa and *Candidatus* Nitrospira Nitrificans were detected in our samples, but in relatively low abundance (<0.3%, 3,082 RPKM on average), consistent with the abundance in other activated sludge systems (Chao et al., 2016; Gonzalez-Martinez et al., 2016). The abundance of Comammox *Nitrospira* was also decreased by ultrasound (Figure 2B; Supplementary Figure S6).

Nitrite oxidation by all nitrite-oxidizing microorganisms is conducted by nitrite oxidoreductase enzyme that has α and β subunits, and the corresponding genes *nxrA* and *nxrB* have been used to quantify nitrite-oxidizing bacteria in the environment (Daims et al., 2016). In our study, both *nxrA* (Wilcoxon test *p* = 0.029) and *nxrB* (Wilcoxon test *p* = 0.039) genes were significantly decreased by 22.2 and 77.4%, respectively (Figure 2C; Supplementary Figure S5). Overall, the above results confirm that ultrasonic treatment was able to select out nitrite-oxidizing microbes and stabilize partial nitrification.

Compared with other partial nitrification methods which either introduce inhibitory substances or control substrate concentration, ultrasound has a unique mechanical effect. In our study, the genes involved in cell membrane components were also significantly reduced after ultrasound treatment (Supplementary Figure S7), suggesting that surviving microorganisms may share common features in cell membrane structures such as appendages, flagella and fimbriae. Indeed, many of the enriched bacteria in our reactor have the ability to form cellular structures with a damping effect similar to that of the appendages (Supplementary Table S6). These appendages may interfere with the inactivation effect by weakening the mechanical effects induced by the ultrasonic cavitation effect (Gao et al., 2016b). For example, both *Nitrospira* and *Nitrolancea* in this study lack intracytoplasmic membrane (ICM) (Garrity et al., 2001), which instead is a typical structure of NOB species of *Nitrobacter* and *Nitrococcus* and AOB species of *Nitrosomonas* (Garrity et al., 2005). The absence of ICM in NOB might suggest that ultrasound killed bacteria like NOB by disrupting the membrane of NOB.

### Functional Profiles

**Figure 3:**
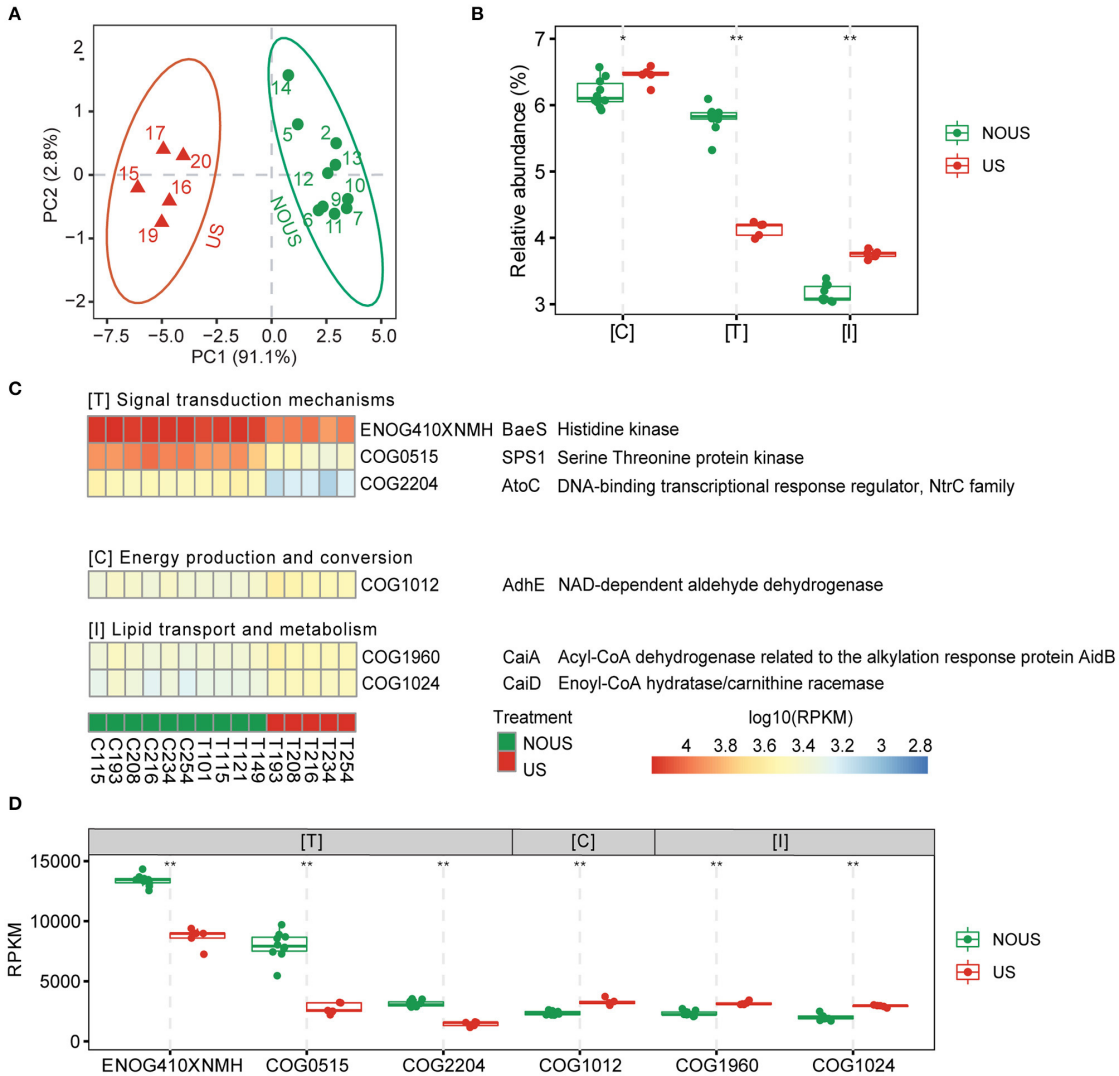
Functional differences driven by ultrasonic treatment. **(A)** Principle component analysis (PCA) of orthologous group abundances matrix, which showed a similar cluster with the phylogenetic profile. Samples were clustered into ultrasonic treatment (US) and without ultrasonic treatment (NOUS) groups. **(B)** Comparison of the ortholog group (COG) function categories whose abundance significantly changed. The most affected functions of all microorganisms when exposed to ultrasound based on Linear Discriminant Analysis (LDA) of the ortholog groups (COG) annotated functions that were significantly enriched in respective groups (*p* < 0.05, |LDA| > 3.5). **(C)** Heatmap of the COGs that contributed to principal components of the community. The abundance of ortholog groups in each sample was scaled to log10 of reads per kilobase per million mapped reads (RPKM) for the color scale of the heatmap. **(D)** Comparison of NOG using Wilcoxon test. (*p < 0.05; **p < 0.01).

The functional composition and their relationship with the community structure were analyzed for further investigation of ultrasonic partial nitrification. PCA of the functional gene abundance matrices divided the 15 samples into two groups (Figure 3A), one with ultrasonication (US) and the other without (NOUS). The two groups were identical to those derived from the species abundance matrix (Figure 1C), reflecting the consistency of the community structure in terms of function and species.

The result of LDA, which considered both significance and effect size, showed that, of all functions, the signal transduction mechanism was decreased the most with ultrasonic partial nitrification, while energy production and conversion and lipid transport and metabolism functions increased slightly (Figure 3B). Among the COGs that led to differences between the two groups (Supplementary Figure S8), the abundance of genes encoding histidine kinase (BaeS, ENOG410XNMH), serine/threonine protein kinase (SPS1, COG0515) and enzymes involved in phosphorelay signal transduction (AtoC, COG2204) were significantly decreased by ultrasound (Figures 3C,D). For energy production and conversion as well as lipid transport and metabolism functions, three COGs including NAD-dependent aldehyde dehydrogenase (AdhE, COG1012), acyl-CoA dehydrogenase related to the alkylation response protein AdiB (CaiA, COG 1960), enoyl-CoA hydratase/carnitine racemase (CaiD, COG 1024) increased (Figures 3C,D). Considering that NOB *Nitrospira* was the most abundant species in the entire community of our reactor, the decline in signal transduction mechanism may be related to the decrease in NOB abundance. Correlation network analysis of species and functions also showed that *Nitrospira* was significantly positively correlated with signaling mechanisms (Supplementary Figure S9).

The two NOB species, *Nitrospira* and *Nitrolancea*, in our study both have extracellular membrane vesicles with sizes up to 100 nm, which are different from other NOB species (Nowka et al., 2015). Membrane vesicles can mediate intercellular communication in some Gram-negative bacteria involved in signal transduction (Mashburn and Whiteley, 2005). In our reactor, the disruption of vesicles may lead to a reduction of signal transduction, which plays multiple roles in wastewater treatment, such as nitrogen removal and biofilm formation (Ge et al., 2015).

In contrast, microorganisms that did not decrease or even increase, such as AOA and AOB, were mainly associated with COGs involving energy production and conversion and lipid transport and metabolism functions (Graphical Abstract). A similar increase in enzymes involved in the metabolism and energy production of amino acids, fatty acids, and carbohydrates was also found in a study in which Nitrosomonas was the dominant species in the community (Laloo et al., 2018), further confirming a relationship between AOB tolerance and these functions.

## Conclusion

In this study, we explored the mechanism of ultrasonic partial nitrification by analyzing taxonomic and functional variations of microbial communities during the establishment and stabilization of the process. NOB species of *Nitrospira, Nitrolancea*, and Comammox *Nitrospira* were all decreased by ultrasonic treatment, while a typical phylum Proteobacteria that AOB *Nitrosomonas* genus belongs to was retained. Metagenomic analysis and PCR of archaeal *amoA* gene showed that AOA species *Nitrososphaerale* was enriched and became more abundant than AOB during partial nitrification. Statistical analysis of functional genes showed that the decrease of *Nitrospira* was highly correlated with signal transduction, more specifically with genes encoding histidine kinase and serine/threonine protein kinase. On the other hand, genes involved in the production of energy from lipid and carbohydrates increased during ultrasonic treatment. The decrease of genes involved in cell membranes suggested that the membrane structure of cells may play a role in the overall changing of nitrifier communities due to the mechanical effect of ultrasonic treatment. These findings in this study provided deep insight into the microbial ecology of activated sludge during partial nitrification, which is promotive for improving shortcut nitrogen removal techniques and achieving sustainable development in the future wastewater treatment plant.

## Data Availability Statement

The data presented in the study are deposited in the NCBI SRA repository, accession number PRJNA792740.

## Author Contributions

YX: conceptualization, data curation, formal analysis, visualization, methodology, writing—original draft preparation, and funding acquisition. MZ: methodology and data curation. SW: data curation and formal analysis. YL: supervision, project administration, writing—reviewing and editing, and funding acquisition. XH: supervision, project administration, and funding acquisition. All authors contributed to the article and approved the submitted version.

## Funding

This work was supported by the State Key Joint Laboratory of Environment Simulation and Pollution Control (22Y03ESPCT).

## Conflict of Interest

The authors declare that the research was conducted in the absence of any commercial or financial relationships that could be construed as a potential conflict of interest.

## Publisher's Note

All claims expressed in this article are solely those of the authors and do not necessarily represent those of their affiliated organizations, or those of the publisher, the editors and the reviewers. Any product that may be evaluated in this article, or claim that may be made by its manufacturer, is not guaranteed or endorsed by the publisher.
