# Corrigendum: Changes in the species and functional composition of activated sludge communities revealed mechanisms of partial nitrification established by ultrasonication

**DOI:** 10.3389/fmicb.2022.1006638

**Published:** 2022-08-16

**Authors:** Yu Xue, Min Zheng, Shuang Wu, Yanchen Liu, Xia Huang

**Affiliations:** ^1^State Key Joint Laboratory of Environment Simulation and Pollution Control, School of Environment, Tsinghua University, Beijing, China; ^2^Australian Centre for Water and Environmental Biotechnology, The University of Queensland, St. Lucia, QLD, Australia

**Keywords:** wastewater treatment, partial nitrification, microbial community, metagenomics, ultrasonic treatment

In the published article, there was an error in the **Funding** statement. “This work was supported by the National Natural Science Foundation of China (No. 51708326), the Major Science and Technology Program for Water Pollution Control and Treatment of China (Nos. 2017ZX07103007 and 2018ZX07111006), and the Tsinghua University Initiative Scientific Research Program (No. 2018Z02ALB01).” The correct **Funding** statement appears below.

## Funding

This work was supported by the State Key Joint Laboratory of Environment Simulation and Pollution Control (22Y03ESPCT).

The authors apologize for this error and state that this does not change the scientific conclusions of the article in any way. The original article has been updated.

## Publisher's note

All claims expressed in this article are solely those of the authors and do not necessarily represent those of their affiliated organizations, or those of the publisher, the editors and the reviewers. Any product that may be evaluated in this article, or claim that may be made by its manufacturer, is not guaranteed or endorsed by the publisher.

