# Supplementary material for: Changes in the Species and Functional Composition of Activated Sludge Communities Revealed Mechanisms of Partial Nitrification Established by Ultrasonication
Source: Front Microbiol. 2022 Jul 19;13:960608. doi: 10.3389/fmicb.2022.960608 (PMC9344063; doi:10.3389/fmicb.2022.960608)
Supplement: Supplementary file 2 [file Data_Sheet_1.DOCX]

*Supplementary Information*

**Changes in the species and functional composition of activated sludge communities revealed mechanisms of partial nitrification established by ultrasonication**

Yu Xue ^a^, Min Zheng ^b^, Shuang Wu ^a^, Yanchen Liu ^a,^ *, Xia Huang ^a^

^a^ State Key Joint Laboratory of Environment Simulation and Pollution Control, School of Environment, Tsinghua University, Beijing, China, 100084

^b^ Advanced Centre for Water and Environmental Biotechnology, The University of Queensland, St. Lucia, QLD 4072, Australia

***Corresponding author**

Email address: [liuyc@mail.tsinghua.edu.cn](mailto:liuyc@mail.tsinghua.edu.cn) (Yanchen Liu)

# 1 Supplementary Figure


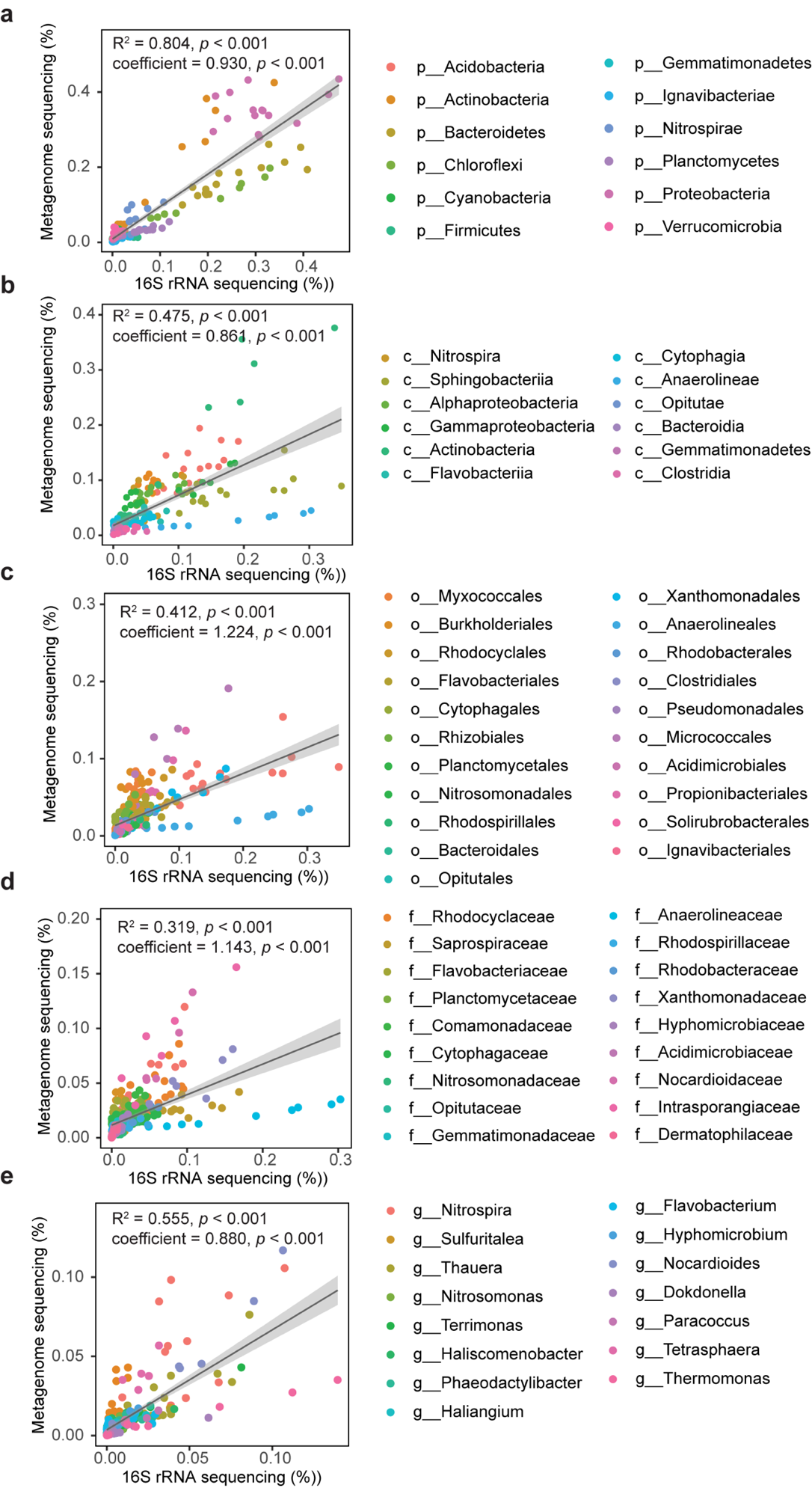


## Fig. S1. Linear correlation of 16S rRNA gene sequencing and metagenomic sequencing.

Comparison of community composition using 16S rRNA sequencing and metagenomic sequencing at various level: (a) phylum level, (b) class level, (c) order level, (d) family level, (e) genus level. Significance test proved linear regression model feasible (*p* < 0.001). Estimated coefficient of the linear model was also presented in each figure.


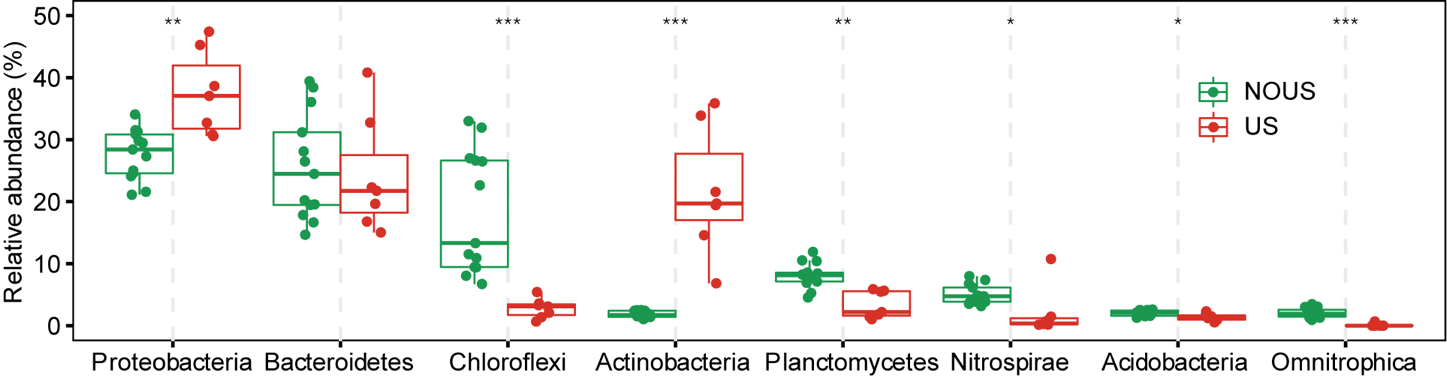


## Fig. S2. Comparison of the abundance of the top eight phyla.

Wilcoxon signed-rank tests between ultrasonic treatment group (US) and non-ultrasonic treatment group (NOUS) were conducted. (*, *p* < 0.05; **, *p* < 0.01; ***, *p* < 0.001).


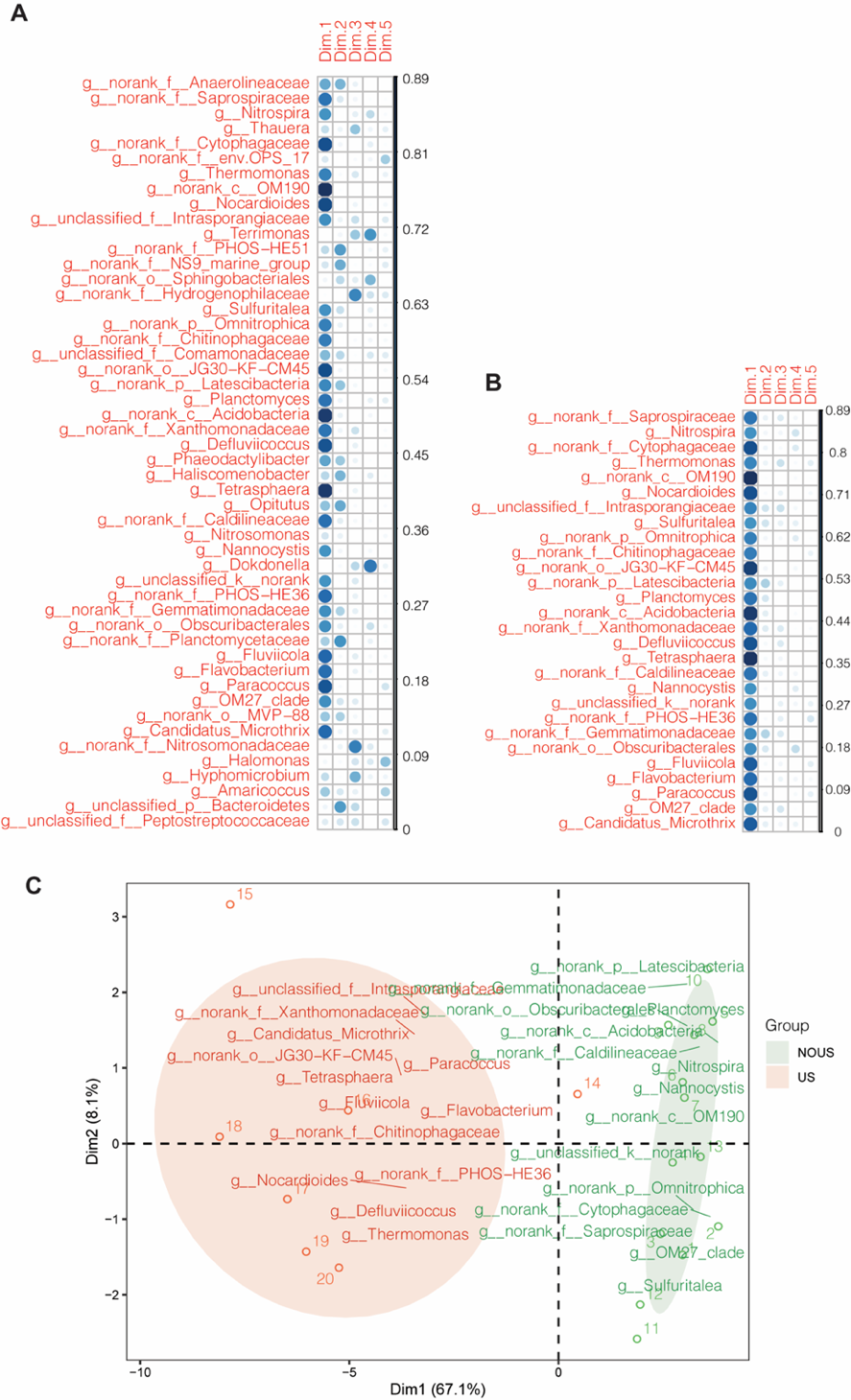


## Fig. S3. Contribution of each genus to each dimension of PCA.

The 50 most abundant genera abundance matrix of each sample were used for analysis. Genera that contribute the most were selected based on cos2 for the genus variable above 0.50. Cos2 (square cosine, squared coordinates) represent the quality of genera variables on factor map.


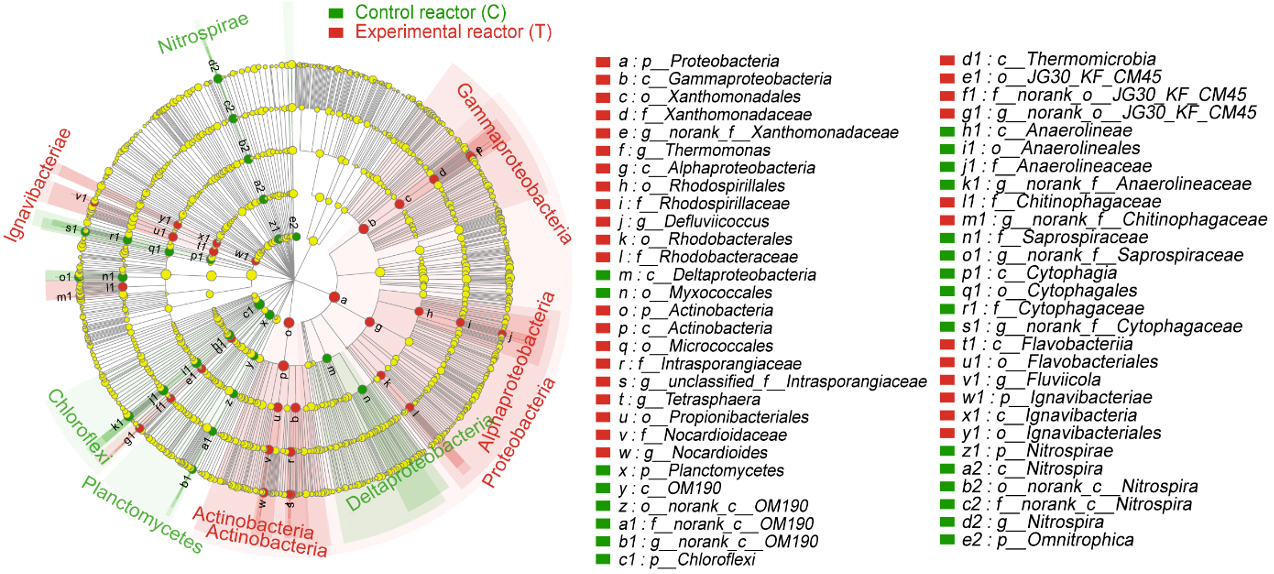


## Fig. S4. Linear discriminant analysis (LDA) of microbial community.

Linear discriminant analysis was conducted for bacterial difference identification in control and experimental reactors. LDA threshold value was 2.5. Differences are represented by the colour of the most abundant class (Green and Red: species that were significantly enriched in respective groups, *p* < 0.05; Yellow: species that were not significantly different in abundance between two groups). The diameter of each circle is proportional to the taxon’s abundance.


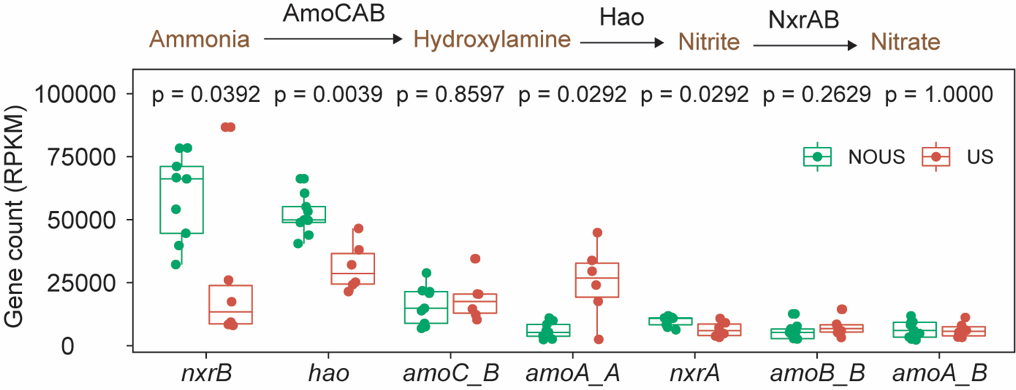


## Fig. S5. Comparison of nitrification gene abundance.

Reads per kilobase per million mapped reads (RPKM) of different genes involved in nitrification including ammonia monooxygenase subunit A of AOA (*amoA*_A), ammonia monooxygenase subunit A, B, and C of AOB (*amoA*_B, *amoB*_B, and *amoC*_B, respectively), hydroxylamine dehydrogenase (*hao*), alpha and beta subunits of nitrite oxidoreductase (*nxrA* and *nxrB*, respectively). Wilcoxon signed-rank tests between ultrasonic treatment group (US) and non-ultrasonic treatment group (NOUS) were conducted.


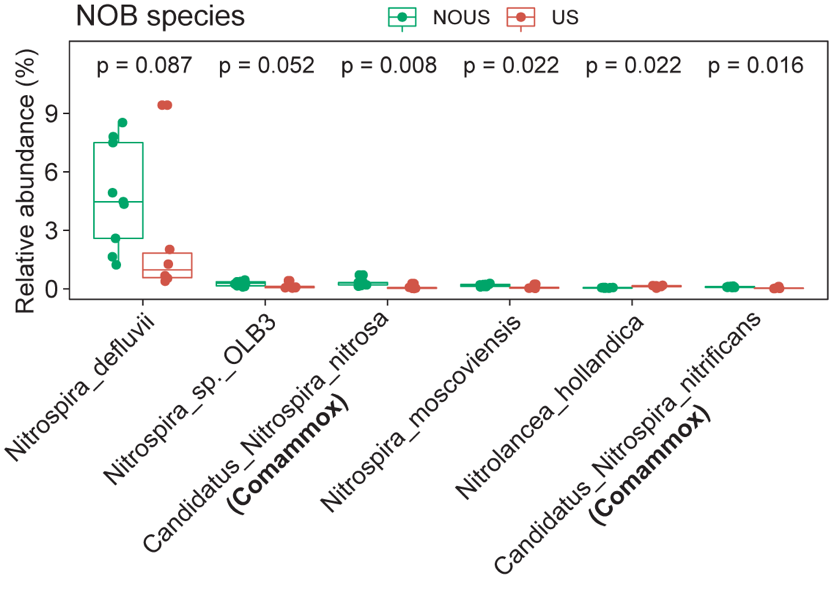


## Fig. S6. Comparison of the abundance of nitrite-oxidizing bacteria species.

Wilcoxon signed-rank tests between ultrasonic treatment group (US) and non-ultrasonic treatment group (NOUS) were conducted.


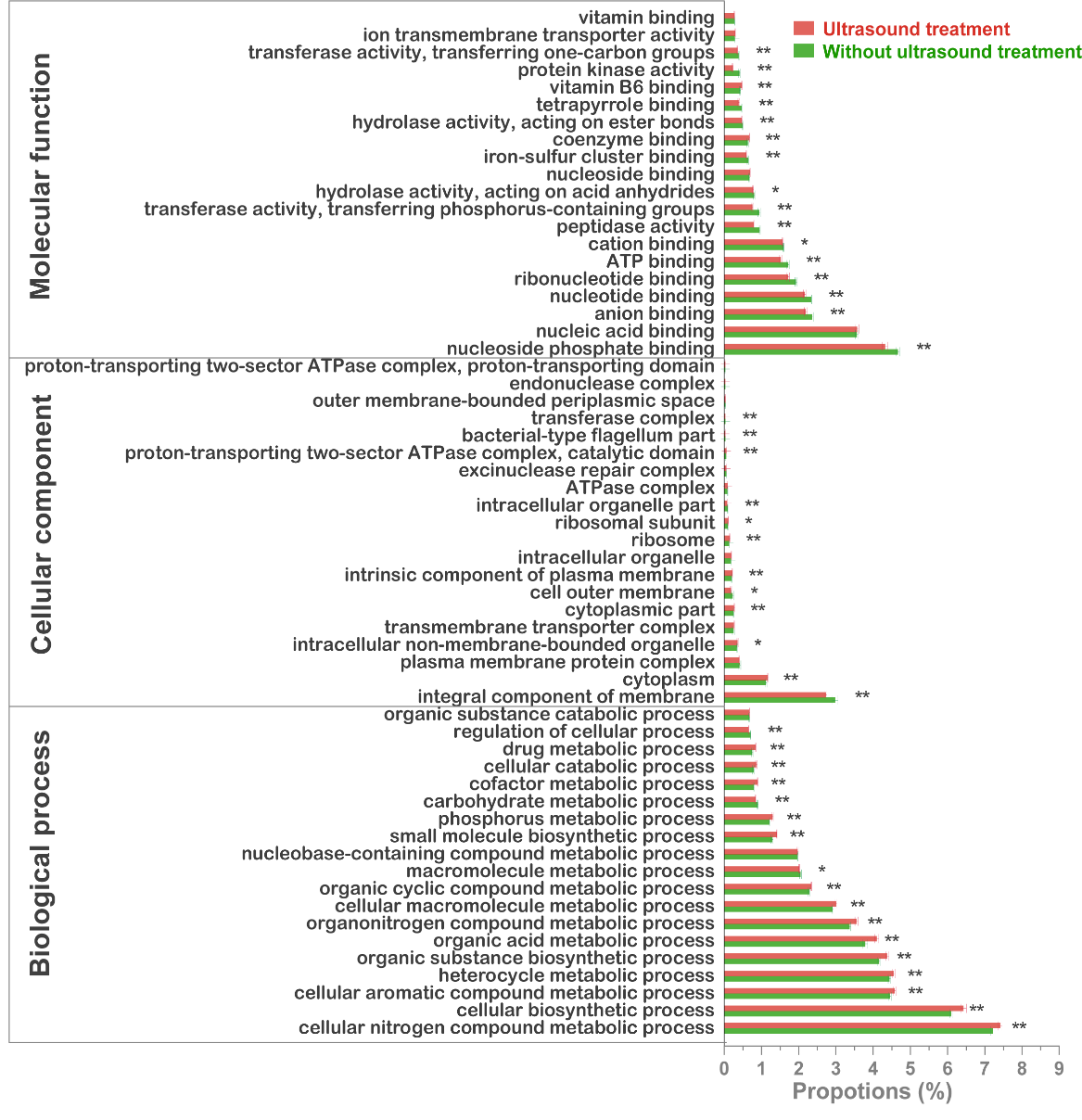


## Fig. S7. Comparison of  Gene Ontology (GO).

The comparison was conducted between ultrasonic treatment group and non-ultrasonic treatment group. Relative abundance of each function was calculated based on reads per kilobase per million mapped reads (RPKM). Wilcoxon signed-rank tests (two-tailed) were used to analyze each comparison within ultrasonic treatment group and non-ultrasonic treatment group. (*, *p* <0.05; **, *p* < 0.01; ***, *p* < 0.001).


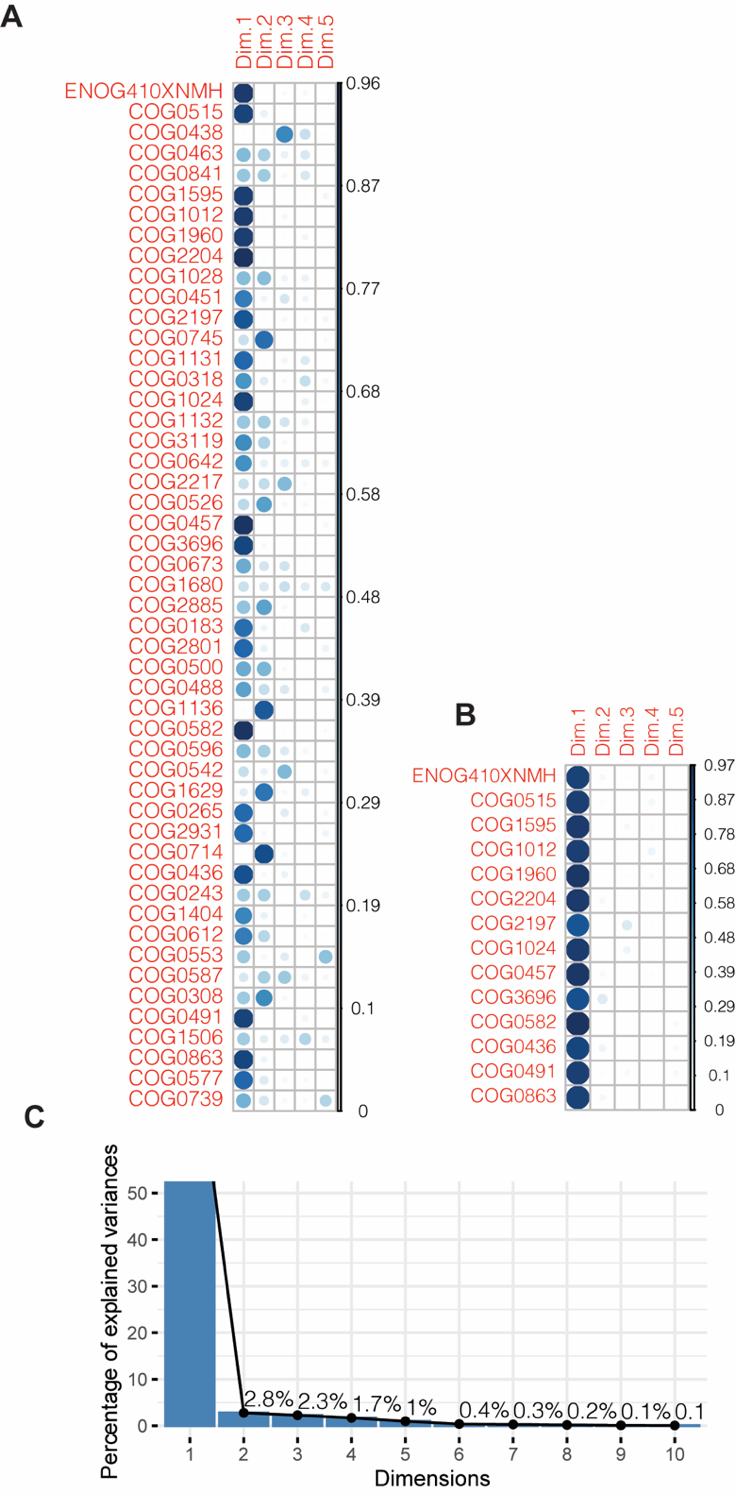


## Fig. S8. Contribution of ortholog groups to each dimension of PCA.

The 30 most abundant ortholog groups (COG) abundance matrix of each sample were used for analysis. Ortholog groups that contribute the most were selected based on cos2 of ortholog group variable above 0.50. Cos2 (square cosine, squared coordinates) represent the quality of ortholog group variable on factor map.


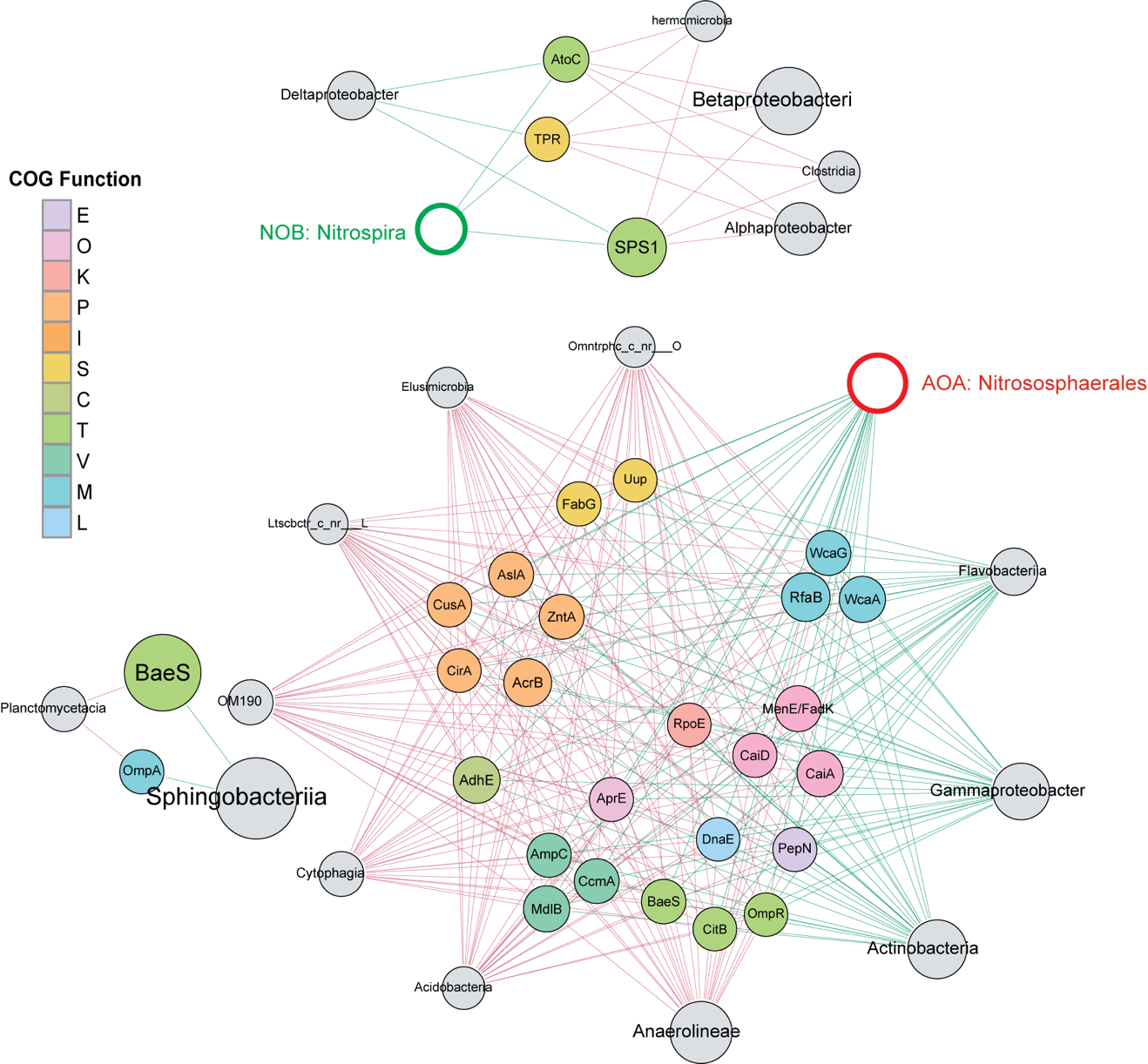


## Fig. S9. Correlation network of microbes and ortholog groups of the whole community.

Absolute spearman correlations above 0.5 were transformed into links between two contributors in the network. The red link represents a positive correlation, while the green link represents a negative correlation. Thicker links represent higher correlations. The bigger of circle size, the more abundant the microbe, which was present at the class level. The microbes that belong to the same phylum or the ortholog groups that belong to the same COG function category were set the same color.
